# Lung adenocarcinoma subtype classification based on contrastive learning model with multimodal integration

**DOI:** 10.1038/s41598-025-13818-2

**Published:** 2025-08-19

**Authors:** Changmiao Wang, Lijian Liu, Chenchen Fan, Yongquan Zhang, Zhijun Mai, Li Li, Zhou Liu, Yuan Tian, Jiahang Hu, Ahmed Elazab

**Affiliations:** 1 Shenzhen Research Institute of Big Data, Shenzhen, China; 2 National Cancer Center, Chinese Academy of Medical Sciences and Peking Union Medical College, National Clinical Research Center for Cancer, Cancer Hospital and Shenzhen Hospital, Shenzhen, China; 3 Zhejiang University of Finance and Economics, Hangzhou, China; 4 The Second Affiliated Hospital, School of Medicine, The Chinese University of Hong Kong, Shenzhen, China; 5 Hongqi Hospital Affiliated to Mudanjiang Medical University, Mudanjiang, China; 6 School of Biomedical Engineering, Shenzhen University, Shenzhen, China

**Keywords:** Lung Adenocarcinoma, Clinical Information, Multimodal Learning, Breast cancer, Cancer imaging

## Abstract

Accurately identifying the stages of lung adenocarcinoma is essential for selecting the most appropriate treatment plans. Nonetheless, this task is complicated due to challenges such as integrating diverse data, similarities among subtypes, and the need to capture contextual features, making precise differentiation difficult. We address these challenges and propose a multimodal deep neural network that integrates computed tomography (CT) images, annotated lesion bounding boxes, and electronic health records. Our model first combines bounding boxes with precise lesion location data and CT scans, generating a richer semantic representation through feature extraction from regions of interest to enhance localization accuracy using a vision transformer module. Beyond imaging data, the model also incorporates clinical information encoded using a fully connected encoder. Features extracted from both CT and clinical data are optimized for cosine similarity using a contrastive language-image pre-training module, ensuring they are cohesively integrated. In addition, we introduce an attention-based feature fusion module that harmonizes these features into a unified representation to fuse features from different modalities further. This integrated feature set is then fed into a classifier that effectively distinguishes among the three types of adenocarcinomas. Finally, we employ focal loss to mitigate the effects of unbalanced classes and contrastive learning loss to enhance feature representation and improve the model’s performance. Our experiments on public and proprietary datasets demonstrate the efficiency of our model, achieving a superior validation accuracy of 81.42% and an area under the curve of 0.9120. These results significantly outperform recent multimodal classification approaches. The code is available at https://github.com/fancccc/LungCancerDC.

## Introduction

Lung cancer stands as one of the most prevalent cancers worldwide and is the leading cause of cancer-related deaths among men. It ranks as the second leading cause of cancer deaths among women, with its incidence rising each year^1,2^. Lung cancer is primarily categorized into two types: small cell lung cancer (SCLC) and non-small cell lung cancer (NSCLC), with NSCLC accounting for about 85% of all cases^3^. Within NSCLC, lung adenocarcinoma (LUAD) emerges as the most common subtype^4^. LUAD is further divided based on histological characteristics and the degree of tumor invasion into three categories: adenocarcinoma in situ (AIS), minimally invasive adenocarcinoma (MIA), and invasive adenocarcinoma (IA)^5,6^. AIS is localized within the alveoli, and early detection often leads to successful surgical intervention, resulting in a high cure rate and a positive prognosis. Likewise, recognizing MIA at an early stage enables timely treatment, which can halt its progression to more aggressive invasive adenocarcinoma, thus enhancing survival rates. Even though IA is already invasive, early detection significantly improves patient outcomes by reducing the risk of recurrence through surgical and adjuvant therapies. Therefore, accurately differentiating among these types of adenocarcinoma is essential for healthcare professionals to ensure effective early diagnosis and treatment.

Low-dose computed tomography (CT) is the main method for early screening and diagnosis of lung cancer^7^. It enables doctors to assess vital tumor characteristics such as size, shape, location, metastasis, and heterogeneity, which are crucial for identifying the type of lung cancer. However, this process depends heavily on the expertise of experienced physicians, which can lead to variability in diagnostic opinions. Furthermore, the subtle histological differences among adenocarcinoma subtypes make distinguishing them based solely on visual CT features difficult. To address these challenges, diagnosing adenocarcinoma subtypes frequently requires integrating clinical information, including patients’ electronic health records (EHR). Physicians often combine this clinical data with CT findings to enhance diagnostic accuracy^1^. While this approach improves the precision of diagnoses, it also significantly increases the workload for healthcare professionals and reduces efficiency.

The advancement of machine learning and radiomics has facilitated automated diagnosis using CT images^8^. Radiomics involves extracting histological and morphological features from these images based on established knowledge and specific rules, with these features often demonstrating strong associations with clinical outcomes. However, raw images need pre-processing to identify regions of interest (ROI), such as cancerous areas, which typically involves manual annotation by skilled radiologists or segmentation algorithms. Once features are extracted, they usually undergo further selection to retain only the most relevant ones, often employing statistical methods like LASSO and principal component analysis^9^. Subsequently, machine learning models, including supervised methods like random forest^10^, support vector machine^11^, and generalized linear models^12^, or unsupervised methods like k-nearest neighbors^13^ are applied^14^. Nonetheless, radiomics-based approaches sometimes fail to fully utilize information from CT images because significant data is lost during feature extraction, including regions adjacent to lesions that might correlate with the disease type. Additionally, the feature selection process can lead to further information loss, and traditional machine learning models, with their limited capacity for parameters, often face challenges in achieving optimal model fitting and performance.

In the past decade, developments in deep learning technologies have helped address challenges in the early detection of lung cancer^15^. Convolutional neural networks (CNNs), such as the ResNet series^16^, have greatly enhanced the accuracy of recognizing features in CT scans. ResNet addresses the vanishing gradient problem in deep network training by using residual connections, which allows for the creation of deeper networks with improved accuracy and performance. More recently, the Transformer architecture^17^, especially the Vision Transformer (ViT)^18^, has outperformed CNN-based models while also reducing computational demands. Additionally, the contrastive language-image pre-training (CLIP) model^19^ has shown significant promise as a vision-language tool. Deep learning automates the extraction of visual features from CT scans, serving as a valuable quantification tool. This automation facilitates the identification of various lesions, offering faster and more precise diagnostic support for physicians^20,21^, and alleviates the workload on radiologists^22^. In multi-view, Zhou et al.^23^ introduced an ensemble multi-view 3D CNN model that excels in risk stratification of lung adenocarcinoma, Luo et al.^24^ proposed cross-aligned representation learning, even surpassing experienced doctors in evaluating invasive adenocarcinoma. Despite these advancements, accurately differentiating cancer subtypes remains challenging. Wang et al.^25^ combined different models, which enhanced preoperative diagnosis. Recent neurological studies have significantly advanced the development of Spiking Neural Networks (SNNs). Despite these advancements, the learning methods for SNNs are not yet fully understood ^26^. In addressing this gap, Agarwal et al. introduced a dual Encoder-Decoder framework specifically designed for processing CT images ^27^. Building on this work, they also developed a multi-scale dual-channel feature embedding decoder aimed at improving biomedical image segmentation ^28^. Furthermore, Mandal et al. explored optimization techniques by implementing a Real Coded Genetic Algorithm to effectively reduce errors ^29^. Existing models often struggle with classification accuracy because lesions are confined to small areas within CT scans, which contain considerable redundant information. Furthermore, effectively distinguishing cancer subtypes necessitates integrating both visual data and patient clinical information, a task that traditional network models find difficult to manage.

The development of multimodal approaches has also significantly enhanced the capabilities of deep learning in the medical field. Yang et al.^30^ examined the CT features of lung adenocarcinoma across different demographic groups and found that these features vary between genders and age groups. Studies by Yu et al.^31^ and Guo et al.^32^ have demonstrated notable advancements. Wang et al.^33^ proposed an approach that integrates multiple imaging modalities but only visional modal. Integrating features from multiple sources often yields superior results compared to relying on a single feature set^34^. Vale-Silva et al.^35^ introduced the MultiSurv, a model applied across 33 different cancer types, which extracts features from visual data using CNN models like ResNeXt-50^36^ while processing clinical data through fully connected networks. The model employs a max-pooling method to combine these feature representations. However, this straightforward fusion might overlook important information within each modality and their interactions. Similarly, TMSS^37^ utilizes a ViT encoder to process multimodal data by integrating CT, positron emission tomography (PET) scans, and EHR for tasks such as segmentation and prognosis. Another approach, CLIP-Lung^38^, uses a textual knowledge-guided framework to predict lung nodule malignancy. The LLM-guided model^39^ employs large language models to analyze clinical notes and align them with image data. Although this model effectively integrates multimodal data and examines the relationships between different features, it struggles with information redundancy in large CT datasets, potentially overlooking critical histological features in lesion areas.

This paper introduces a novel approach, a CLIP-Enhanced Multimodal Fusion Network (CMMFNet), designed to distinguish between lung adenocarcinoma subtypes effectively. Our method integrates multi-sized CT scans, corresponding lesion boundary boxes, and EHR. We utilize the CLIP module^19^ to extract features and calculate cosine distances between modalities, thereby enhancing contrastive loss optimization and improving the alignment of visual and textual information. In extracting features from CT images, bounding boxes are used to delineate lesion areas, enhancing the image data’s semantic representation. This enables the acquisition of ROI-based features, which are focused on the ROIs that contain the lesion, allowing the model to capture more meaningful and context-relevant information. To further refine this process, we introduce a hybrid attention mechanism for deep feature fusion in two stages. The first stage employs a multi-head self-attention mechanism to capture feature representations both within and between modalities, enhancing information coupling and interconnectivity. The second stage applies a Channel attention mechanism to emphasize the relative importance of different modality features, optimizing the feature fusion process with greater precision. This two-stage attention mechanism significantly enhances the completeness and semantic depth of multimodal feature representations, thereby improving the model’s ability to differentiate among various cancer subtypes. The main contributions of our work are outlined as follows:

- We enhance the semantic representation of CT images by leveraging bounding boxes around lesions to focus feature extraction on the ROI, which improves localization accuracy and ensures more precise lesion characterization.
- We propose a two-stage attention mechanism that integrates multi-head self-attention and channel attention, effectively coupling features across different modalities and assigning appropriate weights to each one for improved model performance.
- Our model generates a rich contextual feature representation by incorporating CT scans of varying resolutions, enabling more accurate differentiation of cancer subtypes.
- We evaluate the proposed CMMFNet model on public and proprietary datasets to verify its superiority over state-of-the-art methods.

## Method

**Fig. 1:**
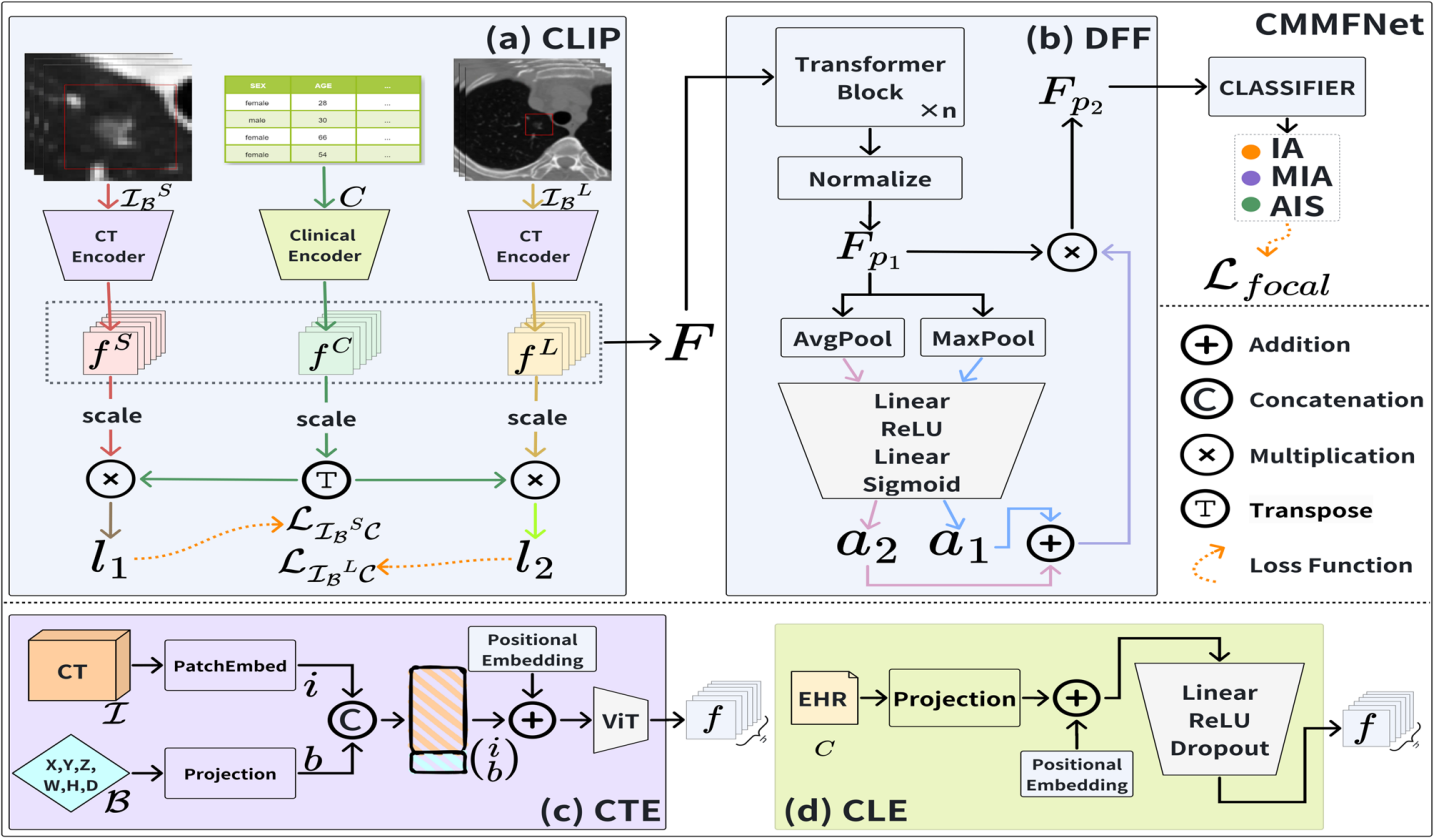
The overall architecture of the proposed **CMMFNet**. It contains **(a) CLIP** and **(b) DFF** module. CT with bounding boxes are processed by an encoder based on the ViT module, **(c) CTE**, to capture and integrate multi-scale image information. EHR data is encoded using an FC-Base module, **(d) CLE**, to extract and integrate clinical text features.

This study introduces the CMMFNet, a multimodal fusion network designed to diagnose lung adenocarcinoma by integrating CT images, positional data, and clinical information. As depicted in Fig. 1, the process begins with the merger of three key inputs: $\{\mathcal {I_B}^S, \mathcal {I_B}^L, \mathcal {C}\}$. Here, $\mathcal {I_B}^S$ represents the small-sized images cropped from the original CT scans using lesion bounding boxes $\{\mathcal {I}^S, \mathcal {B}^S\}$, $\mathcal {I_B}^L$ are the corresponding large-sized CT images, and $\mathcal {C}$ represents the clinical data. These inputs are processed through the CLIP module, which extracts features from these diverse modalities. To enhance feature alignment, we calculate the cosine similarity between the extracted features, which informs the contrastive loss calculation. The resulting fused features, referred to as $\textbf{F}$, are further refined through the Deep Fusion Framework (DFF), an attention-based module that facilitates deeper integration of the features. Ultimately, the integrated features are passed through a classification head to produce the diagnostic outcome. In the following subsection, we discuss each component of the CMMFNet in detail.

### Multimodal feature extraction module

We employ a CLIP-based architecture to extract features from CT scans and clinical data, effectively. This architecture processes two sizes of CT images, each associated with lesion-bounding boxes, alongside structured clinical information. This process results in the extraction of features denoted as $f^S, f^C, and f^L$, representing each modality, respectively. The primary components of this model are as follows.

**The CT Encoder (CTE)** module, depicted in Fig. 1(c), is built on the ViT framework. Initially, a CT image $\mathcal {I}$ is processed through a 3D patch embedding technique. For an image with dimensions $w \times h \times d$, it is divided into patches using a patch size of 8, resulting in $\lceil \frac{w}{8} \rceil \times \lceil \frac{h}{8} \rceil \times \lceil \frac{d}{8} \rceil$ patches. Each patch is embedded in a vector space of $h$ dimensional, forming a matrix $i_k \in \mathbb {R}^{1 \times h}$, where $i=\left\{ i_k \right\} _{k=1}^{N}$.

The bounding box $\mathcal {B}$, centered at $(x, y, z)$ with dimensions $(w, h, d)$, is adjusted through a fully connected layer to match this vector space, resulting in a matrix $b \in \mathbb {R}^{1 \times h}$. The matrices $i$ and $b$ are concatenated into $$\begin{pmatrix} \begin{array}{c} i \\ b \end{array} \end{pmatrix} \in \mathbb {R}^{(1+N) \times h}$$. Positional encoding with learnable parameters is then applied to this concatenated matrix. The matrix is subsequently processed through a standard 12-layer ViT module for encoding. The output from the final layer of this module is averaged across the channel dimension to produce the final output.

**The Clinical Encoder (CLE)** module, shown in Fig. 1(d), utilizes a fully connected network to encode clinical information. This process starts with EHR data, represented as $\mathcal {C} \in \mathbb {R}^{1 \times l}$. The EHR data is initially transformed through a fully connected layer to reshape it to $\mathbb {R}^{l \times h}$. Positional encoding with learnable parameters is then applied to enhance the model’s comprehension of the data’s structural relationships and uncover latent interactions among features. The transformed vector is subsequently processed through another fully connected layer, adjusting its dimensions to produce $f^C \in \mathbb {R}^{1 \times h}$. This final transformation aligns the clinical feature length with that of the visual modality feature.

**Contrastive learning:** The encoded feature vectors $f$ are processed through two distinct pathways. In the first pathway, these vectors are directly concatenated and passed to the next stages of the network, represented as $$\text {F} = \begin{pmatrix} f^S&f^C&f^L \end{pmatrix}^{\top }$$. In the second pathway, features from each modality are normalized using the following equations:

1 $$\begin{aligned} z_1 = \frac{f^S}{\Vert f^S\Vert } ,\quad z_2 = \frac{f^C}{\Vert f^C\Vert } ,\quad z_3 = \frac{f^L}{\Vert f^L\Vert }. \end{aligned}$$

This normalization results in dimensionally consistent feature vectors $z_1, z_2, z_3$. To compute the contrastive loss, the cosine similarities between these features are calculated and scaled by a temperature parameter: $\alpha = \exp \left( \log \frac{1}{0.07}\right)$. The cosine similarities between image and clinical features are given by:

2 $$\begin{aligned} l_1= \alpha \cdot z_1 \cdot {z_2}^\top ,\quad l_2= \alpha \cdot z_3 \cdot {z_2}^\top . \end{aligned}$$

The logits $l_1$ and $l_2$ derived from both image modalities and clinical data are then used to calculate the contrastive loss $\mathcal {L}_{\mathcal {I_B}^S\mathcal {C}}, \mathcal {L}_{\mathcal {I_B}^L\mathcal {C}}$, as detailed in Section Dynamic loss regulation.

### Deep features fusion module

To achieve feature fusion, we develop an attention-based module, referred to as **DFF**, as shown in Fig. 1(b). Initially, the feature $F \in \mathbb {R}^{B \times 3 \times h}$ undergoes a two-step sequential process using the attention-based fusion modules. This approach effectively integrates the features. The fused features are then passed to the classification head, which produces the final category output.

**Phase 1:** To integrate the concatenated multimodal features, we treat them as a unified entity and utilize an $n$-layer Transformer block to identify intrinsic correlations among these features. Each Transformer block is composed of multi-head self-attention and a feed-forward network. The input features pass through multiple attention layers, enabling the model to capture global dependencies within the data. After processing through the final Transformer block, layer normalization is applied to the output to ensure stable training.

The forward process of this module is structured as follows: The input feature tensor $F$ is passed through a series of Transformer blocks. Within each block, multi-head self-attention is employed to extract contextual representations from the input. After processing through each block, the hidden state $F_i$ is preserved for further analysis. This sequence of operations can be summarized as follows:

3 $$\begin{aligned} F_{i+1} = T(F_i), \end{aligned}$$

where $T(\theta )$ represents the comprehensive transformation within a Transformer block. This transformation is detailed as follows:

4 $$\begin{aligned} Q&= \theta W_Q ,\quad K = \theta W_K ,\quad V = \theta W_V, \end{aligned}$$

5 $$\begin{aligned} A(Q, K, V)&= \text {softmax}\left( \frac{Q K^\top }{\sqrt{d_k}}\right) V, \end{aligned}$$

6 $$\begin{aligned} T(\theta )&= \theta + \{A(\theta _i W_Q, \theta _i W_k, \theta _i W_v)\}_{i=1}^{m}W_o, \end{aligned}$$

where $W_Q, W_K, W_V \in \mathbb {R}^{d \times d_k}$ are the projection matrices, and $d_k$ is the dimension of each attention head. Here, $\theta _i$ refers to one of the $m$ attention heads, and the relation $d_k = \frac{h}{m}$ holds. The matrix $W_o$ is the output projection matrix.

The final output of the Transformer block, $F_n$, is normalized to ensure consistency:

7 $$\begin{aligned} F_{p_1} = \frac{F_n}{\Vert F_n\Vert }. \end{aligned}$$

This normalized output, $F_{p_1}$, is then prepared for the subsequent phase, Phase 2.

**Phase 2:** To improve feature representation, we incorporate a channel attention mechanism that selectively highlights important channels. This approach starts by performing pooling operations to gather information from each channel, which is then processed through a fully connected layer to determine the attention weights. During pooling, we use both average pooling and max pooling to capture spatial information across channels, offering two different perspectives on channel significance. These pooled features are then passed into a two-layer fully connected network to compute the attention weights. A reduction ratio is applied to manage the dimensionality of the intermediate layer, and the attention weights are normalized using a sigmoid function to maintain values between 0 and 1. The operations are defined as follows:

8 $$\begin{aligned} AP(\mathbf {\theta })_{i,j}&= \frac{1}{|R|} \sum _{(p,q) \in R} \mathbf {\theta }_{i+p, j+q}, \end{aligned}$$

9 $$\begin{aligned} MP(\mathbf {\theta })_{i,j}&= \max _{(p,q) \in R} \mathbf {\theta }_{i+p, j+q}, \end{aligned}$$

10 $$\begin{aligned} g_1(\theta )&= \sigma ({W_2} \cdot \text {ReLU}({W_1} \cdot AP(\theta ))), \end{aligned}$$

11 $$\begin{aligned} g_2(\theta )&= \sigma ({W_2} \cdot \text {ReLU}({W_1} \cdot MP(\theta ))), \end{aligned}$$

where $AP$ and $MP$ represent the average and max pooling processes, respectively, and $R$ specifies the pooling region indices. The weights $W_1 \in \mathbb {R}^{C \times C/r}$ and $W_2 \in \mathbb {R}^{C/r \times C}$ are learnable parameters of the fully connected layers, with $r$ as the reduction ratio. The sigmoid function $\sigma (\cdot )$ is used for normalization.

The final channel attention weights are computed by summing the weights from both pooling operations and applying them to the input feature map, resulting in the weighted feature $F_{p_2}$:

12 $$\begin{aligned} F_{p_2} = F_{p_1} \cdot ( g_1(F_{p_1}) + g_2(F_{p_1}) ). \end{aligned}$$

This method enables the network to focus on the most informative channels, thereby enhancing overall feature representation by adjusting the input feature map based on the calculated attention weights. We specifically configured the model with 12 Transformer layers and an embedding size of 768. This ViT model was adapted based on the 3D version of MONAI. ^40^

### Dynamic loss regulation

The total loss function utilized in this study consists of two contrastive loss components, $\mathcal {L}_{\mathcal {I_B}^S\mathcal {C}}$ and $\mathcal {L}_{\mathcal {I_B}^L\mathcal {C}}$, along with a focal loss^41^, $\mathcal {L}_{focal}$. The contrastive loss is designed to optimize the cosine distance between different modalities, ensuring that similar representations of image and clinical information are closely aligned. Meanwhile, the focal loss addresses class imbalance by assigning greater weight to difficult-to-classify examples, thereby improving classification performance across imbalanced categories. The combined loss function is expressed as:

13 $$\begin{aligned} \mathcal {L}_{total} = \omega (\mathcal {L}_{\mathcal {I_B}^S\mathcal {C}} + \mathcal {L}_{\mathcal {I_B}^L\mathcal {C}}) + (1-\omega ) \mathcal {L}_{focal}, \end{aligned}$$

where $\mathcal {L}_{\mathcal {I_B}^S\mathcal {C}}$ optimizes the cosine similarity between small-sized CT images and clinical data, and $\mathcal {L}_{\mathcal {I_B}^L\mathcal {C}}$ pertains to large-sized CT images and clinical data. The focal loss $\mathcal {L}_{focal}$ handles class imbalance by computing class-specific losses. The hyperparameter $\omega$ balances the contrastive and focal losses. To dynamically adjust $\omega$, we apply the dynamic loss scaling principle:

14 $$\begin{aligned} \omega = \frac{\mathcal {L}_{\mathcal {I_B}^S\mathcal {C}} + \mathcal {L}_{\mathcal {I_B}^L\mathcal {C}}}{(\mathcal {L}_{\mathcal {I_B}^S\mathcal {C}} + \mathcal {L}_{\mathcal {I_B}^L\mathcal {C}}) + \mathcal {L}_{focal}}. \end{aligned}$$

This dynamic adjustment ensures that if one loss component becomes larger, its weight is reduced, balancing the two loss values.

The contrastive loss is derived from the cross-entropy loss applied to image and text logits. For each image-text logit pair, the cross-entropy loss is independently computed and averaged across the batch. The loss for a batch of $N$ image-text pairs is calculated as follows:

15 $$\begin{aligned} \mathcal {L}_{\mathcal {I_B}^S\mathcal {C}}&= \frac{1}{N} \sum _{i=1}^{N} \left( \frac{1}{2} \left( \mathcal {L}_{\text {CE}}({l_1}_{i}, \textbf{y}_i) + \mathcal {L}_{\text {CE}}({{l_1}^{\text {t}}}_{i}, \textbf{y}_i) \right) \right) , \end{aligned}$$

16 $$\begin{aligned} \mathcal {L}_{\mathcal {I_B}^L\mathcal {C}}&= \frac{1}{N} \sum _{i=1}^{N} \left( \frac{1}{2} \left( \mathcal {L}_{\text {CE}}({l_2}_{i}, \textbf{y}_i) + \mathcal {L}_{\text {CE}}({{l_2}^{\text {t}}}_{i}, \textbf{y}_i) \right) \right) , \end{aligned}$$

where ${l_1}_{i}$ and ${{l_1}^{\text {t}}}_{i}$ are the logits from the image and text models, respectively, and $\textbf{y}_i$ is the true label for the $i$-th image-text pair. The function $\mathcal {L}_{\text {CE}}(l, y)$ represents the cross-entropy loss between logits $l$ and label $y$. This approach ensures equal consideration of image-to-text and text-to-image relationships during training, with the final loss averaged over all pairs, thereby improving alignment between corresponding representations.

To address class imbalance in the dataset, the focal loss extends traditional cross-entropy loss by focusing less on easily classified examples and more on challenging ones. The focal loss is formulated as:

17 $$\begin{aligned} \mathcal {L}_{\text {focal}} = -\frac{1}{N} \sum _{i=1}^{N} \alpha _t (1 - p_t)^\gamma \log (p_t), \end{aligned}$$

where $p_t$ represents the predicted probability for the true class label, $\alpha _t$ acts as a balancing factor for the class, and the parameter $\gamma$ is the focusing parameter. A higher $\gamma$ value increases the emphasis on difficult examples, improving performance in the presence of class imbalance. We set $\alpha _t$ based on the class distribution (e.g., inversely proportional to class frequencies) to help address the imbalance between classes.

## Experiments

### Datasets and implementation

**The open-source LPCD dataset** ^42^, comprises DICOM images from CT and PET-CT scans, featuring bounding boxes that mark tumor locations and include associated clinical data. This dataset consists of 342 lung cancer instances, which are classified into 246 cases of adenocarcinoma, 55 cases of small cell carcinoma, 36 cases of large cell carcinoma, and 5 cases of squamous cell carcinoma. Given the limited number of squamous cell carcinoma instances, the large cell and squamous cell carcinoma categories are combined, resulting in a three-class dataset with the following distributions: 70.7% adenocarcinoma, 17.3% small cell carcinoma, and 12.0% for the merged category.

**The private LUNA-M dataset**, sourced from the Cancer Hospital & Shenzhen Hospital in Shenzhen, China, includes 1,614 cases of lung adenocarcinoma from 1,430 anonymized patients, comprising 545 males and 885 females. Each case contains CT scans, clinical data, and bounding boxes marking tumor locations. Patients’ ages range from 19 to 86, with half between 46 and 64. The dataset is divided into 63 cases of AAH, 299 of AIS, 389 of MIA, and 863 of IA. Due to the low occurrence and pathological similarity of AAH to adenocarcinoma, AAH and AIS are merged into a single category, creating a three-class dataset with 53.5% IA, 24.1% MA, and 22.4% for the combined AAH and AIS. Initial bounding boxes, which provide approximate 2D annotations of the largest lesion cross-section, are refined to accurately define the lesion’s physical location and depth. This refinement results in a 3D bounding box that fully encapsulates the lesion.

**Dataset Processing:** All CT scans initially have dimensions of $512 \times 512 \times D$ in a 3D volume, where $D$ indicates the number of scans. The slice intervals range from 0.625 mm to 5 mm, but all scans are resampled to a uniform resolution of 1 $\textrm{mm}^3$. To ensure the images are within a relevant intensity range, windowing and truncation are applied to the pixel values, restricting them to the range $[-1000, 400]$ HU, a common practice in medical imaging to focus on the most relevant tissue densities. Each lesion is then center-cropped into 3D blocks of sizes $128 \times 128 \times 32$ and $32 \times 32 \times 32$, with bounding boxes adjusted to relative positions. The accuracy of this cropped data is validated using ITK-SNAP^43^. For data augmentation, random flips are applied to the CT scans along each axis (i.e., axial, sagittal, and coronal), enhancing the model’s robustness and generalization capabilities. Corresponding adjustments are made to the bounding boxes to maintain accurate lesion localization after the flip. Additionally, the EHR data undergoes preprocessing by standardizing numerical variables and applying one-hot encoding to categorical variables.

**Implementation Details:** Our experiments were conducted using a 48G NVIDIA A40 GPU with CUDA Version 12.2. The computational setup included Python 3.11 and PyTorch 2.4. To train the model, we employed five-fold cross-validation. This process involved dividing the dataset into five subsets using stratified sampling, where each subset was used once as a validation set while the remaining four subsets were used for training. This approach ensured that class distributions remained consistent across all folds by employing stratified splitting. We employed the Adam optimizer, starting with a learning rate of $10^{-4}$, a weight decay of 0.001, and beta set to (0.9, 0.99) to handle momentum and adjust the learning rate efficiently. Furthermore, the ExponentialLR scheduler was applied to gradually reduce the learning rate during training, using a gamma value of 0.99. During the training phase, we configured the number of epochs to 500 and used a batch size of 24. For the loss function parameters, we set the initial weight of the contrastive loss to 0.5, as specified in Eq. (13). Additionally, in Eq. (17), the $\alpha _t$ hyperparameter for focal loss was adjusted to be inversely proportional to class frequencies, while the $\gamma$ value was fixed at 0.25.

**Evaluation criteria:** The evaluation of all methods was conducted using widely recognized metrics to ensure a fair and comprehensive comparison. These metrics include Accuracy, Precision, Recall, F1-Score, and Area Under the ROC Curve (AUC). These metrics collectively offer a holistic view of the models’ classification performance, ensuring that improvements are not biased towards a single metric but reflect an overall enhancement in prediction quality.

### Comparison with other methods

**Table 1:** Comparison of the proposed method with baseline methods and state-of-the-art approaches on the LPCD and LUNA-M datasets. **Bold** text highlights the best indicator, while underlined text represents the second-best. $\uparrow$ indicates an increase, and $\downarrow$ indicates a decrease.

Method	Data	Accuracy (%)	Precision (%)	Recall (%)	F1-Score (%)	AUC ($10^{-2}$)
ResNet18 (CT32)^16^	LPCD	71.01	23.67	33.33	27.68	50.00
ResNet18 (CT128)^16^		73.91	57.71	38.46	37.05	82.53
ViT (CT32)^18^		71.01	23.67	33.33	27.68	50.00
ViT (CT128)^18^		71.01	23.67	33.33	27.68	64.87
CLIP-Lung^38^		72.46	42.04	49.08	44.95	76.73
TMSS^37^		79.71	76.67	61.59	63.33	79.62
MultiSurv^35^		78.26	49.89	**65.31**	56.46	78.87
LLM-guided^39^		75.36	49.49	55.78	52.29	85.31
**CMMFNet (Ours)**		**81.16** $\uparrow$*1.45*	**87.89** $\uparrow$*11.22*	62.27 $\downarrow$ ***3.04***	**64.23** $\uparrow$*0.90*	**85.67** $\uparrow$*0.36*
ResNet18 (CT32)^16^	LUNA-M	70.59	63.26	63.48	62.81	85.84
ResNet18 (CT128)^16^		66.56	63.04	59.75	59.70	83.86
ViT (CT32)^18^		69.35	61.88	61.81	61.84	80.97
ViT (CT128)^18^		53.56	17.85	33.33	23.25	52.41
CLIP-Lung^38^		71.52	65.05	65.26	64.64	85.60
TMSS^37^		78.95	74.79	76.58	75.45	88.96
MultiSurv^35^		74.92	70.28	68.46	68.72	88.45
LLM-guided^39^		77.09	72.75	73.50	73.05	90.47
**CMMFNet (Ours)**		**81.42** $\uparrow$*2.47*	**78.12** $\uparrow$*3.33*	**79.39** $\uparrow$*2.81*	**78.63** $\uparrow$*3.18*	**91.20** $\uparrow$*0.73*

In our experiments with the LUNA-M and LPCD datasets, as indicated in Table 1, our method consistently surpassed other approaches. The baseline experiments, drawing from previous studies^16,18^, focused on performance analysis, particularly with the LUNA-M dataset. This focus was necessary due to the highly imbalanced class distribution in the LPCD dataset, which led to predictions overly favoring a single dominant class across all samples. ResNet18 showed superior accuracy compared to the ViT model, achieving improvements of 1.24% and 13.00%. We believe this is due to the ability of CNNs to effectively capture subtle lesion regions. In contrast, Transformer-based models like ViT tend to focus on the overall structure of the image, which can limit their effectiveness in highlighting small, localized areas. Further experiments were conducted using images of varying resolutions. Results from the LUNA-M dataset indicated that CT32 images outperformed CT128 images. This finding underscores the negative impact of redundant information outside the lesion areas on network performance. Specifically, using CT32 images resulted in accuracy improvements of 4.03% and 15.79%. These outcomes highlight the critical importance of concentrating on the region of interest (ROI) to enhance the performance of the network.

**Fig. 2:**
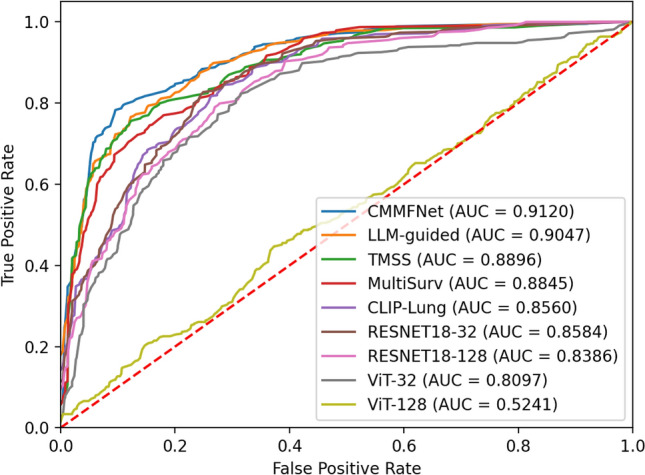
ROC curves of different methods on LUNA-M.

We compared our proposed method to several state-of-the-art multimodal approaches^35,37–39^. While these methods typically use single-size CT images ($32 \times 32 \times 32$) and EHR as inputs, our approach integrates a more comprehensive set of inputs, including dual-size CT images, BBOX, and EHR. On the LUNA-M dataset, our method achieved the highest performance metrics: Accuracy at 81.42%, Precision at 78.12%, Recall at 79.39%, F1-Score at 78.63%, and AUC at 0.9120. These results represent significant improvements over the second-best scores, with increases of 2.47% in Accuracy, 3.33% in Precision, 2.81% in Recall, and 3.18% in F1-Score, in addition to a modest AUC gain of 0.0073. The ROC curves presented in Fig. 2 illustrate that our method, CMMFNet, achieves the most optimal curve position, reflecting its excellent discriminative capacity across various classification thresholds.

Similarly, our method ranked highest on the LPCD dataset, with an Accuracy of 81.16%, Precision of 87.89%, F1-Score of 64.23%, and AUC of 0.8567. These metrics also showed notable improvements over the next best results, with gains of 1.45% in Accuracy, 11.22% in Precision, 0.90% in F1-Score, and 0.0036 in AUC. The results on external datasets such as LPCD^42^ further validate the effectiveness of our proposed method and demonstrate its strong generalization capability, indicating its applicability to different datasets with similar modalities.

Overall, our method achieved leading performance across both datasets. By leveraging state-of-the-art multimodal approaches^35,37–39^, rather than relying solely on CT images, we substantially enhanced accuracy. Furthermore, our proposed method outperformed existing techniques across all evaluated metrics, underscoring its effectiveness and robustness.

### Ablation study

**Table 2:** Results of ablation experiment on LUNA-M. **Bold** text highlights the best indicator. $\uparrow$ indicates an increase, and $\downarrow$ indicates a decrease.

Method	Accuracy (%)	Precision (%)	Recall (%)	F1-Score (%)	AUC ($10^{-2}$)
*CT*32	69.35	61.88	61.81	61.84	80.97
$\mathcal {I_B}^S$	69.04	63.80	63.28	62.64	83.84
*CT*128	53.56	17.85	33.33	23.25	52.41
$\mathcal {I_B}^L$	68.73	63.81	64.29	63.94	82.98
$\mathcal {I_B}^S+\mathcal {I_B}^L$	70.59$\uparrow$*1.24*	64.71$\uparrow$*0.90*	65.13$\uparrow$*0.84*	63.20$\downarrow$ ***0.74***	83.56$\downarrow$ ***0.28***
$\mathcal {C}$	73.99	68.51	69.33	68.57	86.90
$CT32+CT128+\mathcal {C}$	78.02$\uparrow$*4.03*	75.00$\uparrow$*6.49*	76.29$\uparrow$*6.96*	75.22$\uparrow$*6.65*	89.26$\uparrow$*2.36*
$\mathcal {I_B}^S+\mathcal {I_B}^L+\mathcal {C}$	78.64$\uparrow$*0.62*	75.56$\uparrow$*0.56*	76.95$\uparrow$*0.66*	75.95$\uparrow$*0.73*	89.87$\uparrow$*0.61*
$\mathcal {I_B}^S+\mathcal {I_B}^L+\mathcal {C}$ + DFF	**81.42** $\uparrow$*2.78*	**78.12** $\uparrow$*2.56*	**79.39** $\uparrow$*2.44*	**78.63** $\uparrow$*2.68*	**91.20** $\uparrow$*1.33*

**Fig. 3:**
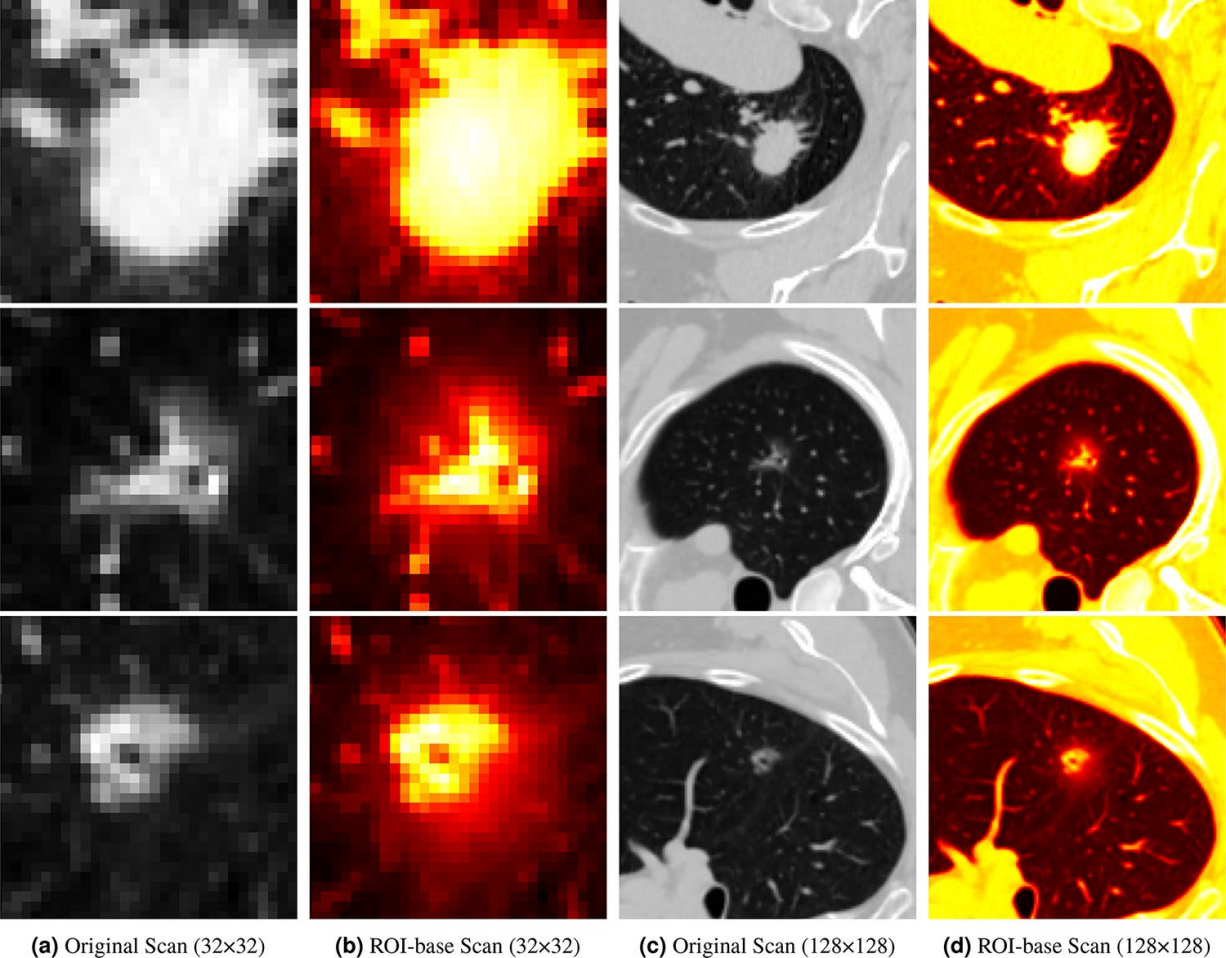
Enhancing semantic representation through ROI-based feature extraction.

To evaluate the effectiveness of each component, we conducted ablation studies on the LUNA-M dataset, with the results presented in Table 2. The findings reveal that utilizing two scales of CT inputs increases accuracy by 1.24% compared to single-scale inputs, highlighting the benefit of multi-scale data in improving model accuracy. For 128-size CT scans, the $\mathcal {I_B}^L$ enhanced with bounding boxes showed improvements of 15.17% in accuracy, 45.96% in precision, 30.96% in recall, 40.69% in F1-Score and 0.3057 in AUC compared to *CT*128, which is using original CT data. This underscores the advantage of incorporating lesion location information for CT enhancements. Our *CT*32, *CT*128 model is designed to focus on cropped lesion regions, or Regions of Interest (RoI), to reduce the inclusion of non-lesion areas. As shown in Table 2, ablation experiments were performed using images with bounding boxes. In contrast, experiments involving *CT*32 and *CT*128 were conducted without the use of bounding boxes. Figure 3 illustrates how ROI-based feature extraction enhances semantic representation across different CT scan sizes, directing more attention to tumor areas.

The integration of clinical information further boosted the model’s performance. Compared to the best-performing single modality ($\mathcal {C}$), incorporating multimodal data resulted in improvements of 4.03% in accuracy, 6.49% in precision, 6.96% in recall, 6.65% in F1-Score, and 0.0236 in AUC. These results highlight the significant advantage of our approach in effectively integrating multimodal data for enhanced predictive performance. The results demonstrate that the combination of $\mathcal {I_B}^S + \mathcal {I_B}^L + \mathcal {C}$ yields a consistent improvement of approximately 0.6% across all metrics compared to $CT32 + CT128 + \mathcal {C}$. This suggests that including images with bounding boxes enhances performance.

The integration of clinical information further boosted the model’s performance. Compared to the best-performing single modality ($\mathcal {C}$), incorporating multimodal data resulted in improvements of 4.03% in accuracy, 6.49% in precision, 6.96% in recall, 6.65% in F1-Score, and 0.0236 in AUC. These results highlight the significant advantage of our approach in effectively integrating multimodal data for enhanced predictive performance. The results show that $\mathcal {I_B}^S + \mathcal {I_B}^L + \mathcal {C}$ leads to a consistent improvement of approximately 0.6% across all metrics compared to $CT32 + CT128 + \mathcal {C}$. This indicates that incorporating images with bounding boxes contributes to the performance boost.

Experiments with the DFF module revealed that attention-based fusion significantly enhanced overall model performance. The model achieved peak accuracy and AUC scores of 81.42% and 0.9120, respectively, with the DFF module contributing improvements of 2.78% in accuracy, 2.56% in precision, 2.44% in recall, 2.68% in F1-Score, and 0.0133 in AUC across all metrics. These results indicate that each component of our approach, including multi-scale input, lesion-aware enhancement, and deep feature fusion, is crucial for enhancing model performance, working synergistically to optimize effectiveness. The AUC primarily assesses the model’s ability to rank positive and negative samples globally, offering a broad evaluation across various classification thresholds. It is not sensitive to class distribution. On the other hand, the DFF module is designed to enhance classification performance at a specific threshold, typically set at 0.5. Consequently, the DFF module markedly improves metrics like accuracy and F1-score, but its influence on the overall ranking performance, as indicated by AUC, is limited. This suggests that while the DFF module strengthens the model’s ability to distinguish samples around the decision threshold, its impact on global ranking capability is relatively minor.

**Student’s t-test: ** Statistical hypothesis testing is crucial for determining whether observed performance improvements are due to actual model enhancements or merely random variations in the data ^44^. The Student’s t-test is a widely used method for comparing the means of two related groups to assess whether their differences are statistically significant. To determine if the improvements in experimental metrics were due to random fluctuations or genuine model enhancements, we conducted a t-test on various result sets. Specifically, we used a paired t-test, suitable for comparing performance metrics before and after model modifications. The calculated *p*-value for our approach in the comparative experiment is 0.0059, and in the ablation experiment, it is 0.0009. Since both values are statistically significant ($p < 0.05$), these results confirm that the observed improvements in performance are indeed due to the proposed model enhancements.

**Fig. 4:**
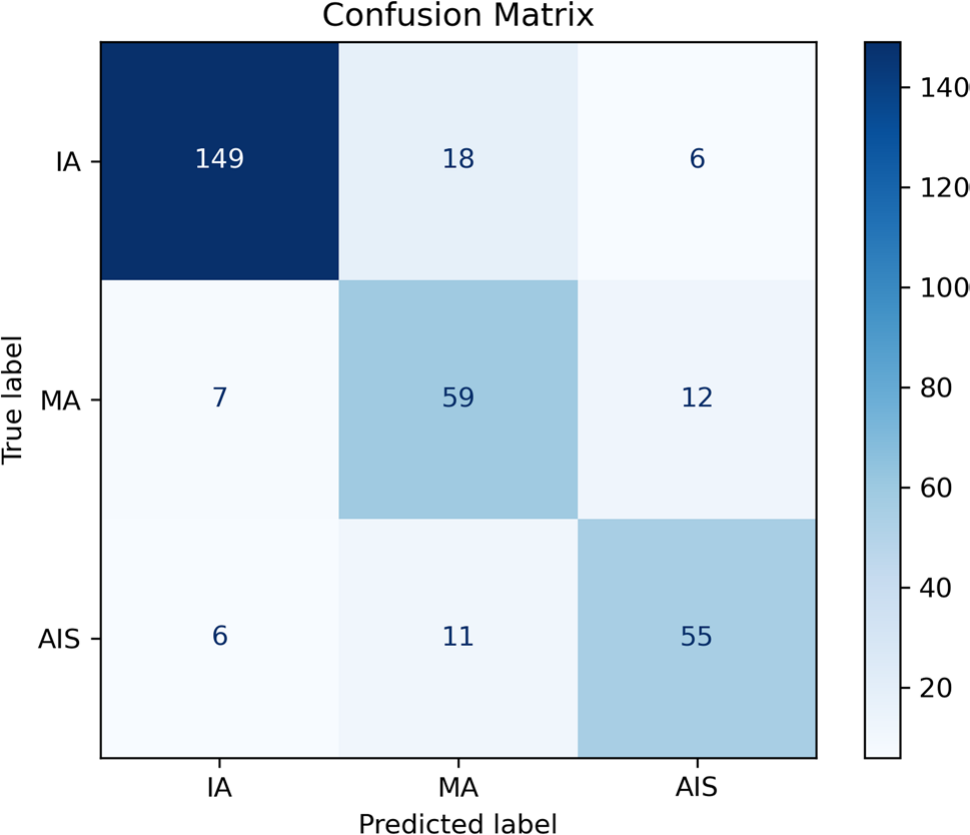
Confusion Matrix on LUNA-M.

The confusion matrix, as depicted in Fig. 4, highlights the model’s performance in predicting lung adenocarcinoma subtypes (IA, MA, AIS). The model demonstrates high accuracy for IA, with 149 cases correctly classified and minimal misclassification into MA (18 cases) and AIS (6 cases). This suggests that the distinct features of IA are effectively captured by the model. However, there is significant confusion between MA and AIS, indicative of their overlapping radiological and pathological characteristics. Specifically, 7 cases of MA were misclassified as IA, and 12 as AIS, while 6 AIS cases were predicted as IA, and 11 as MA. These misclassifications underscore the challenge of distinguishing these subtypes, emphasizing the need for more refined feature extraction or model adjustments. Improving the model’s performance could involve incorporating richer imaging features or leveraging multimodal data, such as clinical and molecular biomarkers. These enhancements would better differentiate between the subtypes and reduce ambiguity, thereby improving the model’s clinical applicability and decision-making reliability.

### Performance analysis

**Table 3:** Comparison of Model Inference Computation Across Different Datasets (Measured by MACs).

Method	MACs (G)	Memory (MiB)	Params (M)	AUC ($10^{-2}$)
ResNet18 (CT32)^16^	**0.48**	28.01	**33.16**	85.84
ResNet18 (CT128)^16^	7.88	34.08	**33.16**	83.86
ViT (CT32)^18^	5.55	**4.43**	85.42	80.97
ViT (CT128)^18^	87.54	141.23	85.42	52.41
CLIP-Lung^38^	58.71	70.19	36.42	85.60
TMSS^37^	87.54	141.23	85.64	88.96
MultiSurv^35^	10.23	34.08	51.29	88.45
LLM-guided^39^	53.22	33.42	37.02	90.47
**CMMFNet (Ours)**	94.11	141.73	284.42	**91.20**

In this study, we evaluate various models based on inference computation, memory usage, and parameter size, as shown in Table 3. We use multiply-accumulate operations (MACs) as the key measure for computational complexity. Memory usage indicates the GPU memory required during inference, and parameter size reflects the model’s complexity.

In the performance comparison, CMMFNet stands out, especially in achieving an AUC score of 91.20, outperforming all other models. Despite having relatively higher computational complexity, memory usage, and parameter count, these attributes contribute to its outstanding performance, particularly in managing complex tasks and enhancing classification accuracy. Models like TMSS and ViT (CT128) are similar to CMMFNet in terms of computational requirements, with MACs and memory usage figures of 87.54G and 141.23MiB, respectively. Nevertheless, they lag behind in AUC scores, achieving only 88.96 and 52.41 compared to CMMFNet’s superior results. Conversely, methods such as ResNet18, which demand less computational power, exhibit poor AUC performance when measured against CMMFNet. Consequently, CMMFNet makes efficient use of computational resources to deliver significant improvements across various performance metrics, underscoring its potential and advantages for real-world applications.

## Conclusion

This paper introduces an innovative deep multimodal network, CMMFNet, designed to classify lung adenocarcinoma subtypes effectively. The network employs the CLIP module for feature extraction and contrastive learning, while the DFF module integrates features from different modalities. Comprehensive experiments on both in-house and public datasets show that CMMFNet outperforms several leading multimodal models across various metrics. By applying ROI-based feature extraction, the network can focus more effectively on the tumor areas, which is especially useful when dealing with adenocarcinoma subtypes that exhibit subtle histological differences. Contrastive learning further enhances the consistency between visual and text-based features, improving the model’s ability to align multimodal information. An attention-based feature fusion mechanism strengthens the coupling of features across different modalities, ensuring that the model can leverage both visual and textual data more effectively.

Nevertheless, this study has several limitations. First, the method relies on initial annotations of lesion bounding boxes for CT cropping and ROI-based feature extraction. Accurate localization of the tumor is crucial for the network to capture both local and global information of the tumor areas. Second, in our approach to EHR data, we focused only on the values, without fully utilizing the semantic meaning of the EHR fields. Different EHR fields contain diverse indicators, and the network’s weights can only be applied to datasets that share the same EHR field structure. For datasets with different field configurations, additional training is required. However, training with additional fields necessitates a large amount of data, which can be a limiting factor. Future work will aim to improve lesion localization, facilitating automated image cropping and enabling the model to capture more detailed lesion features. We also plan to evolve the model into a more comprehensive end-to-end solution. Additionally, we will gather more EHR data to train on specific fields, improving the model’s ability to generate text feature representations in medical terms, ultimately enabling zero-shot transfer similar to CLIP.

In conclusion, the proposed CMMFNet demonstrates reliability in accurately predicting lung adenocarcinoma subtypes. Additionally, its robust performance and integration of multimodal data highlight its potential as a valuable tool to assist doctors in clinical decision-making and treatment planning. In conclusion, the proposed CMMFNet demonstrates reliability in accurately predicting lung adenocarcinoma subtypes. Additionally, its robust performance and integration of multimodal data highlight its potential as a valuable tool to assist doctors in clinical decision-making and treatment planning.

## Data Availability

The datasets used and/or analysed during the current study available from the corresponding author on reasonable request.
